# PD230 The VALUE Project: Multistakeholder Assessment For A Value-Based Prevention of Vaccine-Preventable Lower Respiratory Tract Diseases

**DOI:** 10.1017/S0266462324004495

**Published:** 2025-01-07

**Authors:** Floriana D’Ambrosio, Ada Maida, Ciro Pappalardo, Anna Nisticò, Anna Scardigno, Romina Sezzatini, Walter Ricciardi, Giovanna Elisa Calabro’

## Abstract

**Introduction:**

Lower respiratory tract diseases (LRTDs) pose a public health threat, particularly among vulnerable populations. Vaccination is crucial for disease control, promoting healthy aging, and reducing mortality. However, current immunization programs for adult and the elderly are suboptimal. This project engaged healthcare professionals and citizens to develop guidelines for immunization of adults and the elderly in Italy, with a specific focus on the currently available respiratory syncytial virus (RSV) vaccination.

**Methods:**

The project will include the following three phases: (i) Exploratory phase: a scoping review—involving a search of MEDLINE and institutional websites—to investigate current vaccinations offered to adults and the elderly in Italy for vaccine preventable LRTDs; (ii) Assessment phase: a Delphi process to identify strengths and weaknesses in the current vaccinations offered to adults and the elderly and to evaluate the latest regional and national plans for immunizing the elderly against RSV; and (iii) Policy phase: a consultation with experts to formulate recommendations for the nationwide implementation of vaccination strategies for LRTDs among adults and the elderly, paying particular attention to RSV prevention.

**Results:**

By analyzing current vaccinations offered in Italy, the ongoing project released new evidence and data on vaccine preventable LRTDs to enhance existing vaccination policies and strategies. This aligned a value-based decision-making process with technological innovations in vaccination. Moreover, the involvement of different stakeholders was crucial for identifying health needs and recognizing potential barriers that may hinder adequate vaccination coverage among adult and elderly populations, thus defining priority areas.

**Conclusions:**

Vaccination of adult and elderly populations must be a key component of strategies aimed at promoting the maintenance of good health in the long term. The disease burden of LRTDs and RSV infections is significant and likely underestimated among the elderly. Therefore, adequate prevention strategies are crucial to reducing the national and global burden of these respiratory diseases.

